# A 10-Year Prospective Study of Implant-Based Breast Augmentation and Reconstruction

**Published:** 2018-02-08

**Authors:** Robert El-Haddad, Béatrice Lafarge-Claoue, Charles Garabedian, Sylvain Staub

**Affiliations:** ^a^Centre Chirurgical des Princes, Boulogne Billancourt, France; ^b^Clinique Alphand, Paris, France; ^c^LAMIH, UMR CNRS 8201, UVHC, Valenciennes, France; ^d^IECEP, Boulogne Billancourt, France

**Keywords:** Breast implant, texturation, capsular contracture, cohort study, breast surgery

## Abstract

**Objective:** Observational studies are essential for ensuring patient safety, decreasing complications, and developing better surgical techniques and implants. The primary objective of this study is to demonstrate the safety and efficacy of Sebbin breast implants in both augmentation and reconstruction cohorts. **Methods:** This prospective, multicenter, observational 10-year study conducted in France included 205 patients (385 implants) who underwent breast augmentation (n = 166) or reconstruction (n = 39) with Sebbin round silicone gel implants. Data on patient demographics, surgical details, and complications were collected. **Results:** Median patient age was 39 years; 20.5% of patients were smokers. The augmentation cohort included 166 patients (81.0%); the reconstruction cohort, 39 patients (19.0%). Median implant volume was 280 ml; 91.2% of implants were textured, and 8.8% were smooth. Average patient follow-up was 63 months. The most frequent surgical approach in the Augmentation Cohort was periareolar (72.4%), with 45.5% submuscular and 51.5% subglandular placements. All patients received antibiotic prophylaxis, and postoperative antibiotic therapy was given to 39.5% of patients (average 4.8 days). Drainage was performed in 59.5% of patients (average 2.9 days). Of the reconstruction cohort, 64.1% had preoperative radiotherapy. Nine patients had Baker III/IV capsular contracture (3 bilateral; 4 had a history of radiotherapy) and 7 patients had implant rupture; 41 patients underwent explantation. No cases of double capsule, late seroma, or anaplastic large cell lymphoma occurred. **Conclusions:** This study found an excellent safety profile and very low capsular contracture rate with breast augmentation and reconstruction using Sebbin round silicone gel implants.

According to the data compiled in 2015 by the International Society of Aesthetic Plastic Surgery (ISAPS), breast augmentation is the most frequently performed cosmetic surgical procedure, with figures slightly higher than those of liposuction and blepharoplasty.^1^ Furthermore, the ISAPS estimates that more than 220,000 implant-based breast reconstructions were performed in 2014.

Several high-quality clinical studies were required before the US Food and Drug Administration (FDA) lifted the ban on silicone breast implants. The more recent discovery of the Poly Implant Prothèse (PIP) fraud in 2010 and the increasing number of anaplastic large cell lymphoma (ALCL) diagnoses in patients with breast implants emphasize the ongoing need for observational clinical studies.

Moreover, there have been extensive developments in the field of breast implants focusing mainly on the filling gel and surface textures, but only a limited number of these new implants have been subjected to published clinical evaluation.

Here we report the results of an observational multicenter study conducted in France that monitored 205 patients who underwent breast augmentation or reconstruction using Sebbin round silicone gel-filled breast implants over a 10-year period.

## METHODS

This study started in January 2004 and was conducted by four plastic surgeons with experience in breast augmentation and reconstruction from 4 separate institutions in France. Patients scheduled to receive a breast augmentation or reconstruction with silicone gel-filled breast implants were included, in either the augmentation or reconstruction cohort. The augmentation cohort consisted of patients undergoing surgery due to dissatisfaction with breast size or shape or to an asymmetry or congenital deformity (aplasia, tuberous breast, pectus excavatum). The reconstruction cohort consisted of patients having had a mastectomy for breast cancer. Augmentation and reconstruction patients who had implant replacements were also included in the respective cohorts.

Patients were scheduled for 3- and/or 6-month and annual follow-up visits with their surgeon until 10 years after surgery. Imaging examinations (X-ray and/or ultrasound) were performed at each center per routine and depended on the site.

A case report form was completed at each patient visit. Patient demographics, surgical techniques, and all complications were recorded and collected. Only capsular contractures of Baker grades III and IV were considered as a complication in the statistical analysis.

According to national legislation, the agreement of an ethics committee was not required to conduct this observational study, but the study was conducted in accordance with the Helsinki Declaration and conformed to Good Clinical Practice (ISO 14155).

All data were statistically processed using Statistica software (Tulsa, Okla). The rates of the different complications were computed per patient using the Kaplan-Meier method.

## RESULTS

### Demographic Data

All demographic and surgical data are shown in Table 1. This study included 205 patients (385 implants) with a median age of 39 years (SE = 12.9 years), and 20.5% of patients were smokers. The augmentation cohort included 166 patients (81.0%); reconstruction cohort, 39 patients (19.0%). All the implants used were round; 91.2% were textured, and 8.8% were smooth. The median implant volume was 280 ml.

In augmentation patients, the most frequently used surgical approach was periareolar (72.4% of cases). There were a similar number of submuscular and subglandular placements in this cohort (45.5% and 51.5% of patients, respectively). All patients received antibiotic prophylaxis. Postoperative antibiotic therapy was given to 39.5% of patients and lasted an average of 4.8 days. Drainage was performed on 59.5% of patients and lasted an average of 2.9 days. Of the reconstruction group, 64.1% had preoperative radiotherapy. Thirty-six of muscle-sparing latissimus dorsi (MSLD) flaps were performed in the reconstruction cohort.

### Safety Data

The Kaplan-Meier risks of all recorded complications over the 10-year study period are given in Table 2 with the standard errors. When different durations of follow-up are reported in a study, the Kaplan-Meier analysis provides an estimator (or risk) for the incidence of an event without ignoring patient dropouts. Basically, this analysis takes into account of the last known follow-up visits of the dropped patients. The presented analysis shows cumulative risks.

**Figure 1:**
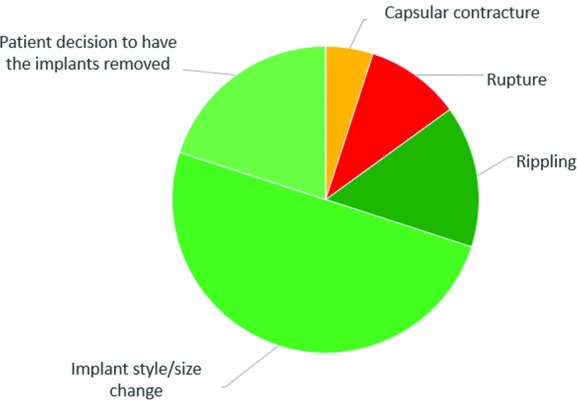
Reasons for implant removal (with or without replacement) on a per-subject basis in the augmentation cohort.

In total, 7 patients had implant rupture (1 was bilateral); 4 of the 7 cases were in the reconstruction cohort. Baker grade III/IV capsular contractures occurred in 9 patients (3 were bilateral); 4 of the 9 patients were in the reconstruction cohort and had undergone preoperative radiotherapy. At least one implant was removed from 41 patients before the end of the study. Figure 1 presents the causes of the 20 explantations in the augmentation cohort. Over these 20 explantations, only 1 and 2 explantations occur after capsular contracture and rupture, respectively. The others were performed for cosmetic reasons (3 explantations for rippling, 10 explantations for style/size change, and 4 patients wanted to remove their implants). Over the 21 other explantations declared in the reconstruction cohort, 66.7% of the explantations were for cosmetic reasons.

There were no cases of double capsule, late seroma, or ALCL. Two cases of axillary adenopathy were observed after 3 and 4 years, and in one case the implant was ruptured. A patient in the reconstruction cohort died due to breast cancer relapse. Three non–breast-located cancers were declared during the follow-up period as well as one case each of fibromyalgia and ankylosing spondylitis.

## DISCUSSION

On review of the scientific literature over the last 10 years, we identified 6 prospective, multicenter studies, each focusing on a specific implant type. They include a 10-year follow-up of patients with silicone gel-filled breast implants whose complications were analyzed by the Kaplan-Meier method.^2^^-^16 Five of these studies correspond to the *Core Studies* requested by the FDA from the three U.S. market manufacturers.^2^^-^14 The sixth one is a study carried out in France on implants from a French manufacturer .^15,16^ Although obvious differences in study design and cohort characteristics preclude a strict straight-forward comparison, comparable monitoring periods, and methods of statistical analysis led us to compare our results with those of these studies.

**Figure 2:**
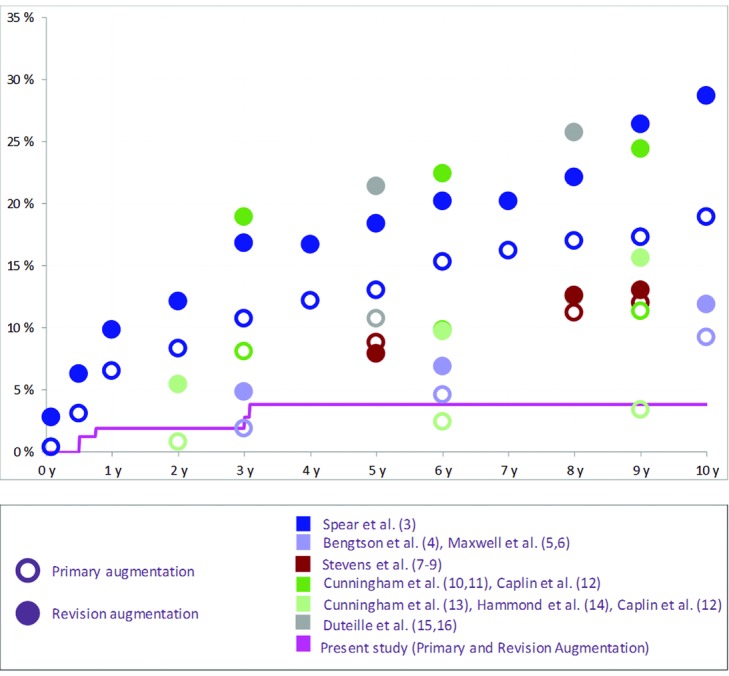
Kaplan-Meier risk of capsular contracture per patient in the Augmentation Cohorts reported on different implant devices.

Interestingly, our study showed a particularly low risk of Baker III/IV capsular contracture. Figure 2 shows the Kaplan-Meier chart for capsular contracture in the augmentation cohorts compared with the aforementioned studies.^2^^-^16

Many recent studies have sought to identify risk factors for capsular contracture after implant-based breast augmentation or reconstruction. By implanting prostheses with shells pre-treated to alter the barrier against perspiration into swine in 2012, Moyer et al^17^ demonstrated that silicone perspiration through the shell increased rates of capsular contracture. The implants used in our study were all fifth-generation cohesive gel implants with a low-bleed barrier.

**Figure 3:**
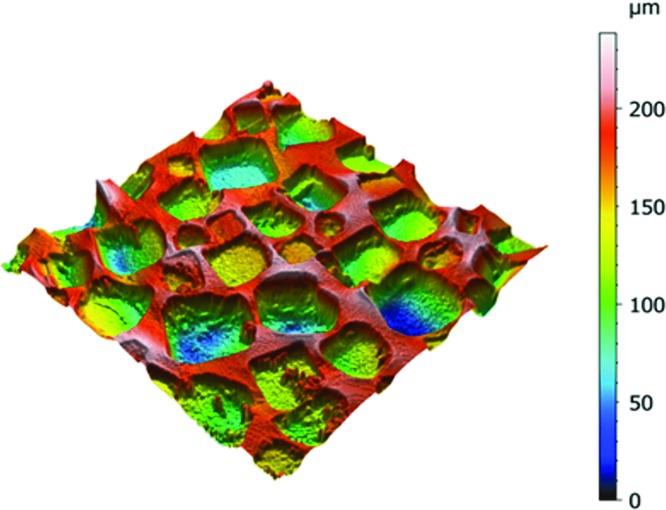
Surface topography of a Sebbin textured round breast implant (X-ray Micro-tomography, LAMIH, Université de Valenciennes, France).

It has also been clearly established that textured breast implants have a lower rate of capsular contracture.^18^^-^24 The clear majority of implants used in this study were textured. The texture of the Sebbin round implants used in this study is obtained using calibrated salt crystals. The surface topography is characterized by cuboidal cavities with a size ranging from 150 to 600 μm and a depth ranging from 100 to 200 μm (Figure 3).

Submuscular placement has also been shown to decrease the rate of capsular contracture.^18^^,^^20^^,^^22^^,^^23^ In our series, approximately half the patients in the augmentation cohort had such placement.

The use of postoperative drainage has been suggested to reduce capsular contracture.^25^^,^^26^ In our study, 51.8% of the patients in the augmentation cohort and 92.3% of the patients in the reconstruction cohort had drainage, with an average duration of 2.9 days. The use of postoperative drainage is unfortunately seldom specified in reports on similarly designed studies.

Recently, the role of biofilm in the onset of capsular contracture has been widely discussed in the literature.^27^^-^29 This could potentially be the cause of the increased capsular contracture rate observed with implants introduced through periareolar incisions,^20^ although the low rate of capsular contracture in our study, which had a relatively high periareolar insertion rate (72.4%) for augmentation patients compared with other similar studies,^2^^-^14 seems to contradict this. In this study, however, all patients received prophylactic antibiotic treatment, and postoperative antibiotic therapy was used in 39.5% of cases. These measures may partially explain the low rates of capsular contracture observed. It is worth noting that, to date, little published data confirms the protective effect of prolonged postoperative oral antibiotics against the occurrence of capsular contracture.

**Figure 4:**
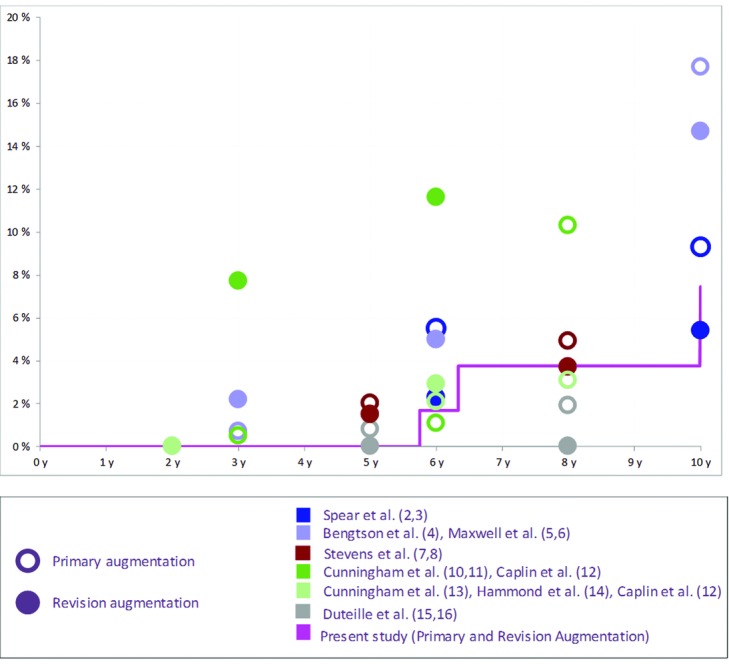
Kaplan-Meier risk of rupture per patient in the Augmentation Cohorts reported on different implant devices.

Figure 4 shows the rupture risk in our study compared with previously published data in the augmentation cohorts.^2^^-^16 It is apparent that the presented results are in the low range of the published data. The presented rupture risk in the reconstruction cohort (21.2%) is higher than the one in the augmentation cohort; however, it is lower than the 10-year risk published by Spear et al^3^ (35.4%). It should be noted, however, that in 4 of the 6 prior studies,^2^^-^6^,^^10^^-^14 rupture rates were analyzed only on subgroups that had undergone magnetic resonance imaging (MRI) monitoring. The MRI techniques have been shown to have superior sensitivity and specificity in detecting breast implant rupture.^30^ This point must therefore be considered when interpreting these results.

**Figure 5:**
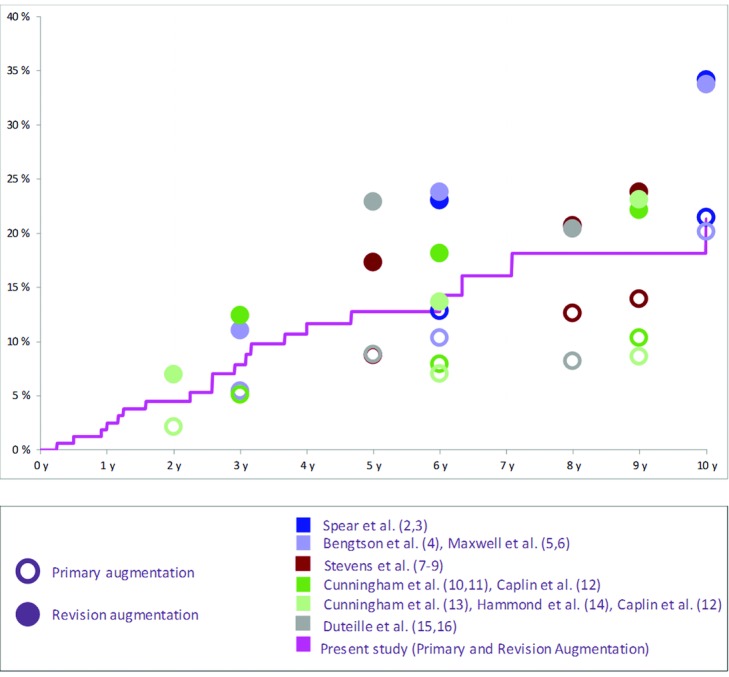
Kaplan-Meier risk of explantation per patient in the Augmentation Cohorts reported on different implant devices.

Figure 5 shows the explantation risk of our study in comparison with previously published data in the augmentation cohorts: they are quite comparable to other studies of similar design.^2^^-^16 It is interesting to note that 85.0% and 66.7% of the explantations were for cosmetic reasons (changing the implant type or volume, correcting asymmetry or rippling, or complying with patient wishes) in the respective augmentation and reconstruction cohorts. In their series of 1788 patients followed over a period of 9 years, Stevens et al^9^ also found that many reoperations occurred for cosmetic reasons (51.4%).

Our results indicate a higher rate of asymmetry when compared to other prospective multicenter series.^2^^-^16 Possible reasons for this are that we did not employ a validated assessment scale and strict reporting of this complication.

Although our study was not designed or powered to statistically evaluate this particular point, our observations are in agreement with the dozens of epidemiologic studies reviewed by Cunningham et al,^10^ (p.275) who “do not support an association between silicone breast implants and systemic disease or symptoms.”

There are obvious limitations to this study. The number of patients was limited in comparison with the previously mentioned studies of comparable design.^2^^-^16 The average follow-up duration in our study was also shorter at 63 months, with 23.4% of patients lost to follow-up after 3 years, 47.8% after 6 years, and 57.0% after 8 years. However, the statistical methodology used in the present article accounts for patients lost to follow-up, having as a consequence larger standard errors.

In conclusion, this 10-year, prospective, multicenter, observational study on Sebbin breast implants demonstrated an excellent safety profile and a very low rate of capsular contracture. There were no cases of late seroma, double capsule, or ALCL.

## Figures and Tables

**Table 1:** Patient demographics, implant device, and surgical data

	Overall	Augmentation cohort	Reconstruction cohort
Patients, n	205	166	39
Implants, n	385	330	55
Median age, y	39	35	52
Median height, cm	166	168	163
Median weight, kg	57	56	59
Median BMI, kg/m²	20	20	22
Device attributes			
Surface characteristics, n (%)			
Smooth	18 (8.8%)	18 (10.8%)	0 (0%)
Textured	187 (91.2%)	148 (89.2%)	39 (100%)
Median device size, cc	280	280	280
Device placement, n (%)			
Submuscular	195 (50.6%)	150 (45.5%)	45 (81.8%)
Subglandular	179 (46.5%)	170 (51.5%)	9 (16.4%)
Unreported	11 (2.9%)	10 (3.0%)	1 (1.8%)
Surgical approach, n (%)			
Periareolar	241 (62.6%)	239 (72.4%)	2 (3.6%)
Inframammary	64 (16.6%)	50 (15.2%)	14 (25.5%)
Transaxillary	32 (8.3%)	32 (9.7%)	0 (0%)
Mastectomy scar	37 (9.6%)	0 (0%)	37 (67.3%)
Other/unreported	11 (2.9%)	9 (2.7%)	2 (3.6%)
Smokers, n (%)	42 (20.5%)	37 (22.3%)	5 (12.8%)
Preoperative radiotherapy, n (%)	26 (12.7%)	1 (0.6%)	25 (64.1%)
Postoperative antibiotics, n (%)	81 (39.5%)	45 (27.1%)	36 (92.3%)
Drainage, n (%)	122 (59.5%)	86 (51.8%)	36 (92.3%)
Follow-up, months	63	60	88

**Table 2:** Kaplan-Meier risks by subject across individual cohorts (standard errors)

		Augmentation	Reconstruction
	Overall, % (SE)	cohort, % (SE)	cohort, % (SE)
Key complications			
Explantation with or without replacement	35.4 (5.3)	21.3 (5.1)	64.1 (8.8)
Rupture	10.9 (4.2)	7.4 (4.4)	21.2 (9.6)
Capsular contracture	5.0 (1.7)	3.8 (1.7)	10.5 (5.0)
Other complications			
Asymmetry	19.5 (3.2)	13.2 (3.0)	47.3 (9.7)
Breast sensation changes	21.9 (3.1)	26.4 (3.6)	2.6 (2.6)
Wrinkling/rippling	16.9 (4.1)	11.5 (3.9)	33.1 (10.5)
Hypertrophic/abnormal scarring	13.2 (2.4)	12.5 (2.6)	16.9 (6.3)
Size change	8.4 (2.9)	8.6 (3.7)	9.0 (5.1)
Swelling	7.3 (1.8)	9.0 (2.2)	0
Hematoma	4.9 (1.5)	6.0 (1.8)	0
Infection	0.5 (0.5)	0.6 (0.6)	0
Breast cyst	3.8 (2.0)	4.8 (2.5)	0
Implant malposition	6.2 (3.0)	5.9 (3.9)	8.8 (6.0)
Calcification	2.1 (1.2)	1.5 (1.1)	3.7 (3.6)
Ptosis	1.0 (1.0)	1.3 (1.3)	0
Granuloma	0.8 (0.8)	0	3.7 (3.6)
